# Method for Analyzing the Molecular and Carbon Isotope Composition of Volatile Hydrocarbons (C_1_–C_9_) in Natural Gas

**DOI:** 10.1155/2018/4512081

**Published:** 2018-12-18

**Authors:** Chunhui Cao, Zhongping Li, Liwu Li, Li Du

**Affiliations:** Key Laboratory of Petroleum Resources, Gansu Province/Key Laboratory of Petroleum Resources Research, Institute of Geology and Geophysics, Chinese Academy of Sciences, Lanzhou 730000, China

## Abstract

Solid-phase microextraction (SPME) coupled with gas chromatography-isotope ratio mass spectrometry (GC-IRMS) has already been applied to collect and identify volatile light hydrocarbons in oil and source rocks. However, this technology has not yet been used to analyze volatile light hydrocarbons in dry gas (natural gas with C_1_/C_2+_ > 95%). In this study, we developed a method to measure the molecular and carbon isotope composition of natural gas using divinylbenzene/carboxen/polydimethylsiloxane (DVB/CAR/PDMS) fiber. This fiber proved to be suitable for extracting C_1_–C_9_ hydrocarbons from natural gas without inducing carbon isotopic fractionation. Notably, the extraction coefficients of the analytes were not the same but rather increased with the increasing carbon number of the hydrocarbons. Nevertheless, we successfully identified 24 hydrocarbons from the in-lab standard natural gas, while also obtaining the carbon isotope composition of C_1_ to C_9_ hydrocarbons with satisfying repeatability. The relative standard deviation (RSD) of the molecular composition data was in the range of 0.06–0.74%, with the RSDs of the carbon isotope composition data not exceeding 1‰. Finally, seven natural gas samples, collected from different sedimentary basins, were successfully analyzed and the stable carbon isotope compositions of C_1_–C_9_ hydrocarbons present in these were determined through this method. Overall, the new approach provides a simple but useful technique to obtain more geochemical information about the source and evolution of natural gas.

## 1. Introduction

Volatile light hydrocarbons (C_1_–C_9_) are important components of crude oil because they possess a large scope of geochemical information that is of great significance to oil and natural gas exploration. Furthermore, these components can be applied to classify source rocks and oil types [1, 2], identify source rock evolution [3–6], study oil-oil or oil-source correlations [1, 7–11], and estimate the thermal maturity of source rocks and crude oil [12–14]. However, restrained by analytical methods, previous studies focused on the measurement of light hydrocarbons only in source rocks and crude oil while not being able to acquire their carbon isotope compositions. Natural gas (particularly dry gas, e.g., shale gas), as opposed to petroleum and source rocks, mainly contains methane and ethane and has extremely low content of C_4+_ hydrocarbons. Nevertheless, despite the scarcity, volatile light hydrocarbons (C_4+_) in natural gas (derived from oil and/or source rocks) and their carbon isotope compositions carry abundant and significant geochemical information. Employing this information assists in the determination of the maturity of natural gas, recognition of gas accumulation suffering from washing or biodegradation, tracing the source of natural gas, and classification of the origin types of natural gas [14–19]. Because natural gas is generated from the oil and/or source rocks, its molecular and carbon isotope composition retains the information about the oil and source rocks [20, 21]. Considering that natural gas, oil, and source rocks contain light hydrocarbons (C_1_–C_9_), we expect that, instead of using an indirect deduction via the molecular and isotope fractionation theory [22–24], a direct study of these components would allow the detection of the correlation between gas, oil, and source rocks [15]. However, implementing this new method to measure the molecular and carbon isotope compositions of light hydrocarbons in natural gas is difficult, due to the limit of detection (LOD) values of analytical instruments.

**Figure 1:**
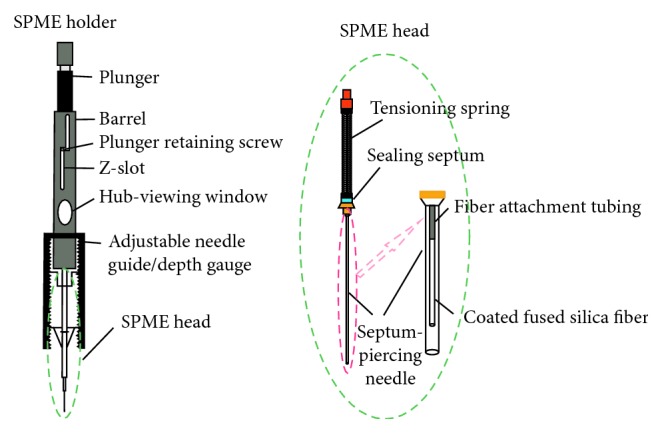
Schematic diagram of the SPME holder and fiber (Supelco Data Sheet No. T713019 A, 1998).

Solid-phase microextraction (SPME) is an innovative, solvent-free sample preparation approach that is fast, economical, and versatile and requires only a small amount of sample. A general SPME device (Figure 1) resembles a modified syringe, consisting of an SPME holder and SPME head with a built-in fiber inside a needle [25]. The SPME holder, which consists of a plunger, stainless steel barrel, and adjustable depth gauge, is usually designed to be used with reusable and replaceable fiber assemblies [26]. The SPME head includes a spring, sealing septum, and piercing needle. The fiber inside the needle is coated with a special polymeric stationary phase, which could concentrate the organic analytes from the sample matrix [26]. Notably, this device has been widely applied in sample pretreatment technology [27], as well as to analyze the hydrogen isotope composition of light hydrocarbons in natural gas [28] and crude oil [29].

In this study, we successfully applied SPME technology to enriched light hydrocarbons (C_1_–C_9_) in natural gas. In addition, we combined SPME with a gas chromatography (GC) or gas chromatography-isotope ratio mass spectrometry (GC-IRMS) system in an effort to measure the molecular and carbon isotope compositions of a series of light hydrocarbons (C_1_–C_9_) in natural gas. Considering that thermogenic natural gas is derived from the cracking process of organic materials (e.g., source rocks and kerogen), the geochemical data of light hydrocarbons in natural gas could definitely provide clues on their source and evolution, which would, in turn, be very relevant to the research of oil/natural gas geochemical scientists.

## 2. Experimental

### 2.1. Materials

For this study, we selected a type of fiber coating with divinylbenzene/carboxen/polydimethylsiloxane (DVB/CAR/PDMS) to enrich the light hydrocarbons in natural gas. An in-lab standard natural gas was employed to test the characteristics of this fiber, and the enrichment conditions were optimized. The in-lab standard natural gas was collected from Ordos Basin, China, but it is not a standard for other laboratories. We analyzed its molecular composition and compared the result with that of three other laboratories to confirm that it mainly contains 24 types of hydrocarbons: methane, ethane, propane, isobutane, *n*-butane, neopentane, isopentane, *n*-pentane, methyl cyclopentane, cyclohexane, 2-methylpentane, 3-methylpentane, *n*-hexane, 2,2/3,3-dimethyl pentane, methyl cyclohexane, 2/3-methyl hexane, 2,3-dimethyl pentane, *n*-heptane, benzene, *n*-octane, methyl benzene, *n*-nonane, ethyl benzene, *p*-xylene, and *o*-xylene. Several separate parallel samples were prepared from the in-lab standard natural gas for the experimental condition optimization. Each sample was collected in a fixed-volume (600 mL) glass bottle that was subsequently sealed with a rubber stopper.

### 2.2. Conditions for the Analysis Instruments

The molecular composition of the gas samples was determined using a gas chromatograph (6890A, Agilent Technologies, USA) equipped with a flame ionization detector. The individual hydrocarbon gas components (C_1_–C_9_) were separated using an AT-Al_2_O_3_ capillary column (50 m × 0.53 mm × 20 *μ*m, Agilent Technologies, USA). The GC oven temperature was adjusted according to the following procedure: 35°C for 3 min, increased to 100°C at a rate of 7°C/min, kept at that temperature for 5 min, 7°C/min ramped to 160°C, kept at that temperature for 10 min, increased to 200°C at a rate of 15°C/min, maintained at that temperature for 30 min, 25°C/min ramped to 220°C, maintained at that temperature for 85 min. The GC injection port temperature was set to 350°C, and the split ratio was 1 : 1. The carrier gas (He) was in a constant-flow mode, with a flow velocity of 2 mL/min.

The stable carbon isotope ratios were measured on a gas chromatography-isotope ratio mass spectrometry system (GC-IRMS, isotope ratio mass spectrometer interfaced with an Agilent 6890A gas chromatograph). The individual hydrocarbon gas components (C_1_–C_9_) were separated on a 6890A gas chromatograph using the instrumental conditions mentioned above. Then, the separated compounds were injected into a combustion furnace for oxidizing at 950°C. The produced H_2_O was removed using a water trap, and CO_2_ was injected into the Delta plus XP mass spectrometer (Thermo-Fisher, Bremen, Germany) for isotopic analysis. An electron impact (EI) ion source was used for the mass spectrometer, with a filament emission current of 1.3 mA and electron energy of 100 eV. CO_2_ (purity ≥99.99%) with a carbon isotopic value of *δ*^13^C_CO2_ = −20.9‰ (±0.5‰) was used as a reference gas. The analytical error in the *δ*^13^C values was <0.4‰ (*n* = 6) for standard natural gas. The stable carbon isotopic values were reported as *δ*-notation in per mil (‰) relative to the Vienna Pee Dee Belemnite (VPDB) standard with a measurement precision for *δ*^13^C of ±0.5‰.

### 2.3. SPME Fiber Selection

The SPME fiber is coated with relatively thin films of several polymeric stationary phases, which are conventionally used as coating materials in chromatography. This film acts like a sponge, concentrating the organic analytes from the sample matrix [26]. The coating on the fiber can consist of a variety of materials, including carbowax template resin, polydimethylsiloxane, polydimethylsiloxane divinylbenzene, polyacrylate, carboxen polydimethylsiloxane, and carbowax divinylbenzene [30, 31]. The carboxen/polydimethylsiloxane (CAR/PDMS) fiber was first used in 2014 to measure the carbon isotope composition of volatile light hydrocarbons in natural gas by combining SPME and GC-IRMS [32]. This research work opened the way to a new application of SPME technology into the natural gas study area. Recently, a type of SPME fiber coating, i.e., divinylbenzene/carboxen/polydimethylsiloxane (DVB/CAR/PDMS), which can be used to extract C_3_–C_20_ volatiles, was found suitable for enriching trace light hydrocarbons in natural gas samples [28, 29]. It has already been applied to the analysis of the hydrogen isotopic composition (*δ*^13^D) of volatile light hydrocarbons in natural gas [28] and crude oil [29]. In this study, DVB/CAR/PDMS fiber was employed to extract trace light hydrocarbon compounds in natural gas samples and analyze their molecular and carbon isotope compositions (*δ*^13^C).

### 2.4. Procedures for the Extraction of Trace Hydrocarbons

**Figure 2:**
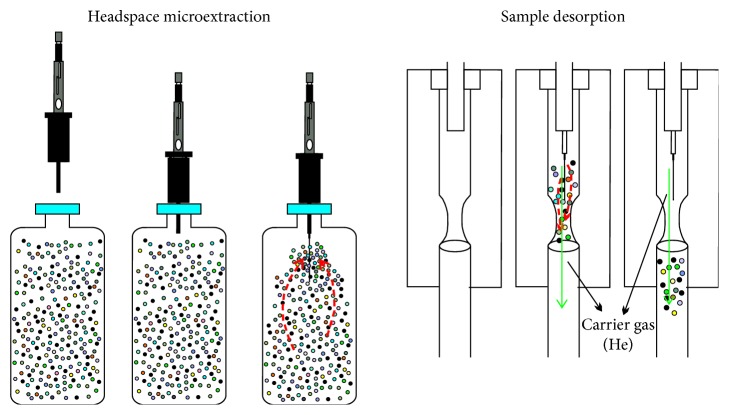
Extraction and desorption processes using the SPME fiber. The dashed red arrows represent the movement of the hydrocarbons in sample, while solid green arrows represent the direction of the carrier gas.

When extracting light hydrocarbons, the septum-piercing needle of the SPME device (Figures 1 and 2) was directly inserted into the glass bottle (600 ml) containing the sample through the rubber stopper. Then, the plunger was pushed to make the coated fiber stretch out of the needle, and the fiber was immersed directly into the natural gas sample to expose the coating to the hydrocarbons to be sampled (Figure 2). This would initiate the absorption of the analyte molecules onto the coating. The transport of analytes from the matrix into the coating begins as soon as the coated fiber has been placed in contact with the sample. After trace hydrocarbons are trapped onto the coating by an equilibrium mechanism, the fiber was retracted into the needle and taken out of the bottle [33]. Then, the fiber can be inserted into the injection port of a GC (or GC-IRMS) (Figure 2) and quickly desorbed by the heat of the port, resulting in a rapid transfer of all absorbed components into GC (or GC-IRMS) for molecular composition (or isotope composition) analysis.

**Figure 3:**
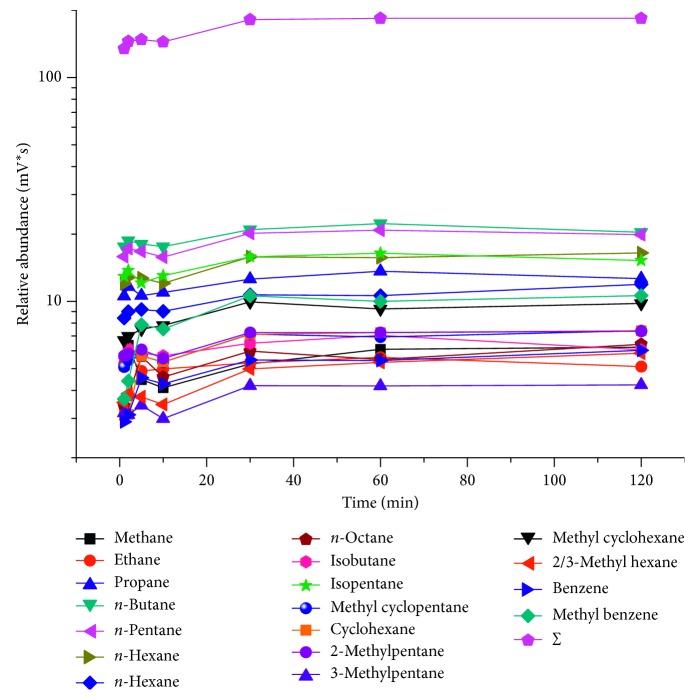
Relationship between the adsorption capacity and adsorption time (desorption time: 2 min).

The extraction time was found to be a critical parameter in the SPME sampling process. The parallel samples of the in-lab standard natural gas were extracted the light hydrocarbons by SPME while applying different extraction times (2, 5, 10, 30, 60, and 120 min). First, DVB/CAR/PDMS fiber was applicated to extract trace light hydrocarbon compounds in one of the in-lab standard natural gas samples at room temperature for 2 min. After finishing trace hydrocarbons extraction by using the SPME device, we have acquired the C_1_-C_9_ hydrocarbons on the DVB/CAR/PDMS fiber. In order to desorb the analytes that were extracted on the fiber and analyze their molecular composition with GC, we should take the SPME device out of the sample bottle and introduce it into the GC injector port where the adsorbed analytes are thermally desorbed at 280°C (most commonly used temperature in GC analysis [34]) and consequently be routed into the GC column for composition analysis [33]. And then, the other five in-lab standard natural gas samples were analyzed using the same procedures, but the extraction time was selected as 5, 10, 30, 60, and 120 min, respectively. As a result, we found that the DVB/CAR/PDMS fiber had an unstable adsorption of hydrocarbons in the first 10 min (Figure 3). The extracted amount of trace hydrocarbons onto the fiber reached a peak value at 2 min, after which it decreased. Between 10 and 30 min, the extracted amount rapidly increased from 144.21 to 181.39 × 10^5^ mV, respectively. After 30 min, the content of extracted analytes did not change much (Figure 3). Therefore, we select to extract the light hydrocarbons at room temperature for 30 min.

### 2.5. Desorption Temperature

**Figure 4:**
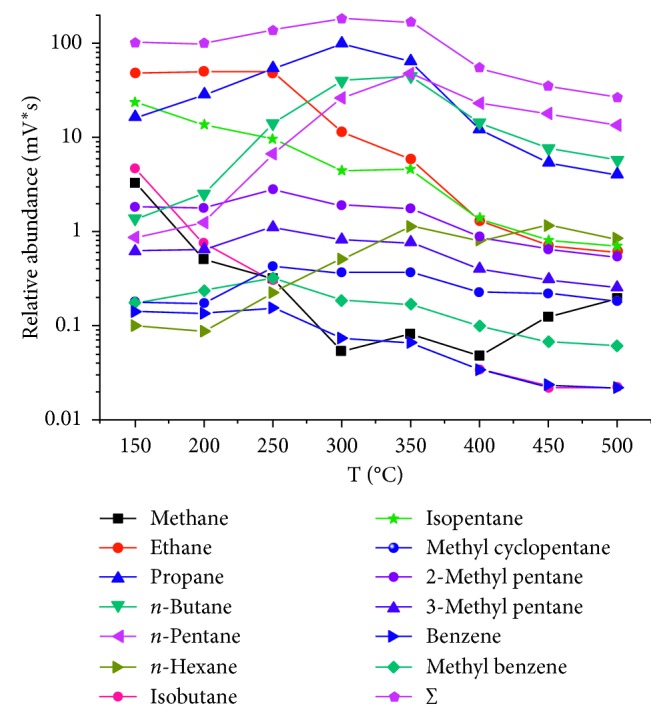
Relationship between the desorption amount and desorption temperature (desorption time: 2 min).

The injection port temperature of GC was found to be a key parameter as it affected the volume of each analyte entering the instruments used for molecular or carbon isotope composition analyses. Different temperatures (150, 200, 250, 300, 350, 400, 450, and 500°C) were selected for the injection port of the GC in order to find the optimal desorption conditions. Eight in-lab standard natural gas samples contained in glass bottles were prepared for the optimal temperature condition test. One of the eight in-lab standard natural gas samples was adsorbed by DVB/CAR/PDMS fiber, thermally desorbed in the injector port of GC at 150°C, and then introduced into the GC column by the carrier gas for molecular composition analysis, respectively. And then, the other seven in-lab standard natural gas samples were analyzed using the same procedures, but the injection port temperature was selected 200, 250, 300, 350, 400, 450, and 500°C, respectively. Notably, most of the compounds displayed a degassing peak at 300 and 350°C (Figure 4). The desorption ratio at 350°C (defined as ∑_desorption amount before 400°C_/∑_desorption amount at each temperature_) of all hydrocarbons exceeded 60%, except for *n*-hexane, which exhibited a ratio of 42.47%. In addition, the amount of each analyte desorbed at 350°C was enough for molecular and carbon isotope composition analyses to be performed. Thus, in order to desorb as much amount of each analyte as possible and ensure that the DVB/CAR/PDMS fiber does not age quickly, 350°C was chosen as the most suitable desorption temperature.

### 2.6. Extraction Performance

**Figure 5:**
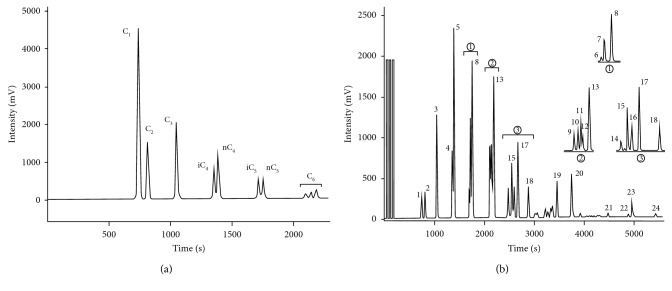
Chromatograms of the light hydrocarbons present in the (a) in-lab standard natural gas and (b) analytes extracted using SPME. 1, Methane; 2, ethane; 3, propane; 4, isobutane; 5, *n*-butane; 6, neopentane; 7, isopentane; 8, *n*-pentane; 9, methyl cyclopentane; 10, cyclohexane; 11, 2-methylpentane; 12, 3-methylpentane; 13, *n*-hexane; 14, 2,2/3,3-dimethyl pentane; 15, methyl cyclohexane; 16, 2/3-methylhexane +2,3-dimethyl pentane; 17, *n*-heptane; 18, benzene; 19, *n*-octane; 20, methylbenzene; 21, *n*-nonane; 22, ethylbenzene; 23, *p*-xylene; 24, *o*-xylene.

By combining SPME and GC, we analyzed the molecular composition of the in-lab standard natural gas using the experimental conditions and operation procedures discussed above. The obtained results were then compared with the ones from the sample without extraction by SPME.  Figure 5(a) shows the chromatogram of the origin in-lab standard natural gas analyzed by GC, while Figure 5(b) displays the chromatogram after the sample was extracted by SPME with the DVB/CAR/PDMS fiber. As can be seen from Figure 5, only C_1_–C_5_ compounds were detected in the natural gas sample without the SPME extraction, whereas, after the extraction by SPME, 24 types of hydrocarbon compounds (C_1_–C_9_) were enriched and measured. The standard deviation (SD) of all compounds, except methylbenzene (0.66%), did not exceed 0.5%. According to the standard spectrum diagram (at the same experimental conditions) obtained with the AT-Al_2_O_3_ capillary column, 24 types of hydrocarbon compounds were identified (Figure 5(b)). After comparing Figures 5(a) and 5(b), we found that the concentrations of methane, ethane, and propane markedly decreased after the extraction, while those of *n*-butane, *n*-pentane, and C_6+_ compounds increased dramatically. These results imply that the DVB/CAR/PDMS fiber exhibited various adsorption abilities on the different hydrocarbon compounds, thus being suitable for extracting C_4+_ hydrocarbons, which are trace compounds in natural gas [15].

**Figure 6:**
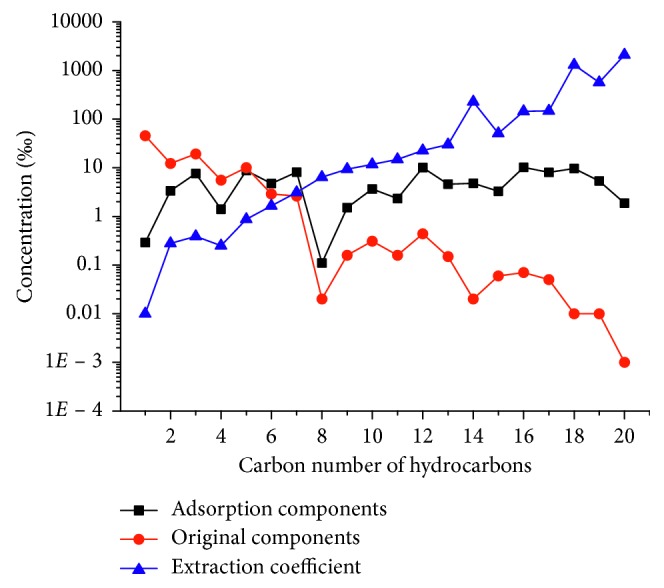
Comparison of the content of each analyte before and after the adsorption by SPME. 1, Methane; 2, ethane; 3, propane; 4, isobutane; 5, *n*-butane; 6, isopentane; 7, *n*-pentane; 8, methyl cyclopentane; 9, cyclohexane; 10, 2-methylpentane; 11, 3-methylpentane; 12, *n*-hexane; 13, methyl cyclohexane; 14, 2/3-methylhexane; 15, *n*-heptane; 16, benzene; 17, *n*-octane; 18, methylbenzene; 19, *n*-nonane; 20, *p*-xylene. Adsorption components: the relative concentration of extracted analytes. Original components: the relative concentration of original analytes. Extraction coefficient: the ratio of each component's relative concentration (vol/%) before and after the extraction.

Although the DVB/CAR/PDMS fiber has a diverse adsorption ability for different compounds, it has a particularly strong extraction ability for C_5_–C_9_ compounds, especially on *n*-alkanes (Figures 5 and 6). As can be seen from the analytical results, the concentration of *n*-hexane, *n*-heptane, *n*-octane, and *n*-nonane increased dramatically. The concentrations of CH_4_, C_2_H_6_, C_3_H_8_, and *n*-C_4_H_10_ in the extracted analytes were lower than those in the original natural gas sample, while the concentration of C_5+_ in natural gas was enriched after the extraction with DVB/CAR/PDMS fiber, to reach the LOD of analytical instruments. Reportedly [15], the concentration of C_1_-C_3_ is higher in natural gas, while that of C_5+_ is usually quite low. Considering that the concentration of C_1_-C_3_ in the extracted analytes was much lower than that of the natural gas sample and the concentration of C_5+_ is raised apparently, we concluded that the fiber had a balancing effect on the hydrocarbons in natural gas. Therefore, the fiber coated with DVB/CAR/PDMS could efficiently overcome the challenge of measuring trace C_5+_ compounds in natural gas.

### 2.7. Extraction Coefficient (*f*)

The extraction coefficient, which is defined as the ratio of each component's relative concentration (vol/%) before and after extraction, respectively, can be used to describe the fiber's enrichment ability for each hydrocarbon. We used the in-lab standard natural gas as a sample to test the enrichment ability of the DVB/CAR/PDMS fiber. The relative concentration of extracted analytes (“Adsorption components” in Figure 6) was measured by SPME+GC, and the relative concentration of original analytes (“Original components” in Figure 6) was analyzed by standard GC techniques. The extraction coefficient (*f*) of each analyte can be described using the following equation:

(1) $$\begin{array}{c} f_{x\;\;}=\frac{C_{x\left(\text{after}\right)}}{C_{x\left(\text{before}\right)}}, \end{array}$$

where *f*_x _ is the extraction coefficient of component x, and *C*_x(before)_ and *C*_x(after)_ are the relative concentrations of component x before and after, respectively, extraction by DVB/CAR/PDMS fiber. As can be seen from Figure 6, the extraction coefficient increased significantly as the carbon number of the analytes became larger. The extraction coefficients of C_1_–C_4_ hydrocarbons were less than 1, which implies that the fiber (coating with DVB/CAR/PDMS) could not enrich these hydrocarbons. In particular, the original concentration of CH_4_ in the in-lab standard natural gas was about 50%. After the extraction, its concentration in the analytes absorbed on the fiber dropped to around 0.3%, thereby affording a CH_4_ extraction coefficient of about 0.006. Nevertheless, the extracted contents of C_1_–C_4_ hydrocarbons by the fiber were enough for these compounds to be detected and for the carbon isotope composition analysis to be successful. Furthermore, after the extraction, the relative concentration (%) of C_5+_ hydrocarbons (trace amount components in the in-lab standard natural gas sample) increased significantly, with their extraction coefficients ranging from 10 to 2000 (Figure 6). Thus, after the extraction, almost every hydrocarbon reached a concentration between 1% and 10%, which indicated that the fiber (coated with DVB/CAR/PDMS) can balance the content of C_1_–C_9_ hydrocarbons in natural gas. Although this characteristic of the DVB/CAR/PDMS fiber allowed the detection of trace components in natural gas, it also caused difficulties in the calculation of the real content of each analyte. In this respect, more tests are needed to develop a mathematical model for the calculation of the real content of trace hydrocarbons in natural gas, especially C_6_–C_8_ components, which are useful for gas source studies. Nevertheless, the content balance effect makes it easier to simultaneously measure the carbon isotope compositions of C_1_–C_9_ hydrocarbons in natural gas.

## 3. Application of Carbon Isotopic Analysis

### 3.1. Stability of Carbon Isotopic Analysis

**Figure 7:**
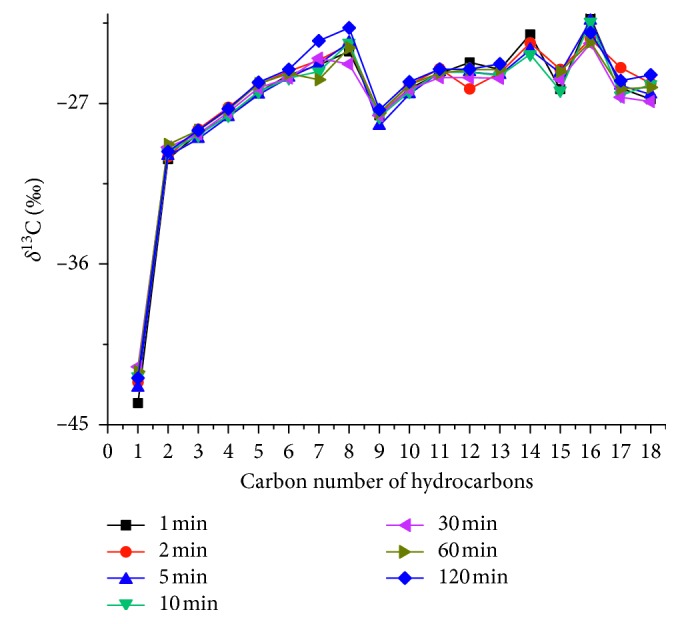
Relationship between the carbon isotope ratios and desorption times of each hydrocarbon. 1, Methane; 2, ethane; 3, propane; 4, isobutane; 5, *n*-butane; 6, isopentane; 7, *n*-pentane; 8, methyl cyclopentane; 9, 2-methylpentane; 10, *n*-hexane; 11, methyl cyclohexane; 12, 2/3-methylhexane; 13, *n*-heptane; 14, benzene; 15, *n*-octane; 16, methylbenzene; 17, *n*-nonane; 18, *p*-xylene.

GC-IRMS paired with SPME was used to analyze the carbon isotope composition of light hydrocarbons in natural gas. Since the analytes underwent physical adsorption and desorption processes before the carbon isotope analysis, several experiments had to be conducted to detect whether carbon isotopic fractionation has occurred during the SPME extraction processes. In this respect, Li et al. [29] have found that the extraction time, extraction temperature, and desorption temperature have no significant effect on *δ*^13^D when using the DVB/CAR/PDMS fiber to analyze hydrocarbons in oil. Therefore, in this work, we selected the following extraction conditions: room temperature for 30 min and desorption at 350°C. In contrast, different desorption durations were selected (1, 2, 5, 10, 30, 60, and 120 min) to detect the effect of the desorption time on *δ*^13^C of each analyte in the in-lab standard natural gas sample. First, one of the in-lab standard natural gas samples (collected in the glass bottle, Section 2.1) was extracted by SPME device using the procedures illustrated in Section 2.4. Then, the SPME needle was introduced into the GC-IRMS injector port where the adsorbed analytes are thermally desorbed for 1 min and routed into the GC column for composition separating. Eventually, individual hydrocarbon gas components (C_1_–C_9_) were oxidized into CO_2_ and H_2_O, and the CO_2_ was let into IRMS for carbon isotope composition analysis. Then, the other six in-lab standard natural gas samples were analyzed using the same procedures, but the duration of desorption was selected 2, 5, 10, 30, 60, and 120 min, respectively. The variation range of the obtained carbon isotopic values of the analytes in the in-lab standard natural gas samples did not exceed 1‰, with a standard deviation not exceeding 0.7‰. As can be seen from Figure 7, the carbon isotope data obtained for each analyte desorbed in different durations overlapped almost perfectly, thus indicating that the desorption time would not cause an apparent isotope fractionation.

### 3.2. Geological Sample Analysis

**Figure 8:**
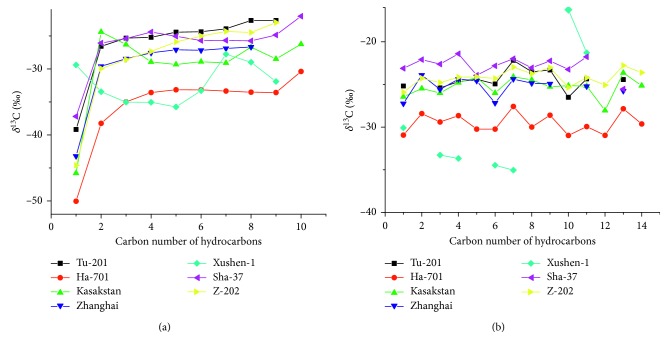
Carbon isotope composition of hydrocarbons in natural gas. (a) 1, Methane; 2, ethane; 3, propane; 4, *n*-butane; 5, *n*-pentane; 6, *n*-hexane; 7, *n*-heptane; 8, *n*-octane; 9, *n*-nonane; 10, benzene. (b) 1, Isobutane; 2, neopentane; 3, isopentane; 4, methyl cyclopentane; 5, cyclohexane; 6, 2-methylpentane; 7, 3-methylpentane; 8, 2,2/3,3-dimethyl pentane; 9, methyl cyclohexane; 10, 2/3-methylhexane +2,3-dimethyl pentane; 11, methylbenzene; 12, ethylbenzene; 13, *p*-xylene; 14, *o*-xylene.

Seven natural gas samples collected from different sedimentary basins (Tarim Basin, Qaidam Basin, Songliao Basin, Huanghua Depression, Tuha Basin, and Bohai Bay Basin) were analyzed using the abovementioned method in an effort to detect the stable carbon isotope compositions of hydrocarbons (C_1_–C_9_) contained therein. The carbon isotope distribution pattern of *n*-alkanes, branched alkanes, and cycloalkanes is shown in Figure 8. More specifically, Figure 8(a) shows the carbon isotope distribution pattern of *n*-alkanes and benzene, while Figure 8(b) displays the patterns for branched alkanes and cycloalkanes. The natural gas form Xushen-1 in Songliao Basin exhibited a heavier *δ*^13^C_1_ value (−29.40‰) than other natural gases and was characterized by a reversed carbon isotopic distribution pattern (*δ*^13^C_1_ > *δ*^13^C_2_ > *δ*^13^C_3_ > *δ*^13^nC_4_ > *δ*^13^nC_5_), which suggested that it is an abiogenic gas [35]. The other natural gases (*δ*^13^C_1_ ranging from –50‰ to –37‰) were recognized as thermal genetic gases or mixtures of thermal genetic gases and abiogenic gas and/or secondary cracking gas.

The thermal genetic methane (*δ*^13^C_1_ = –45.78‰) in the natural gas from Kazakhstan and the partially reversal carbon isotopic distribution pattern (*δ*^13^C_1_ < *δ*^13^C_2_ > *δ*^13^C_3_ > *δ*^13^nC_4_ > *δ*^13^nC_5_), which was due to secondary alterations under high temperature such as oil, gas cracking, and diffusion, indicate the complicate organic thermal evolution process.

## 4. Conclusions

The SPME technique has brilliant application prospects as its specific extraction characteristics provide a simple and useful method to analyze the carbon isotope compositions of trace hydrocarbons (C_1_–C_9_) in natural gas. In this respect, an SPME fiber coating with DVB/CAR/PDMS is suitable for the extraction of C_1_–C_9_ hydrocarbons from natural gas, thereby allowing the extraction of 24 types of hydrocarbons, with an extraction coefficient increasing significantly upon increasing the carbon number. However, the various enrichment abilities of each hydrocarbon make it difficult to calculate the real content of hydrocarbons, while the content balance effect makes it easier to simultaneously measure the carbon isotope composition of C_1_–C_9_ hydrocarbons in natural gas.

The herein presented analysis method has an excellent repeatability and high precision. Carbon isotope stability experiments of trace hydrocarbons proved that the desorption time did not induce any isotopic fractionation. Furthermore, this method was applied to seven natural gas samples in an effort to determine the carbon isotope compositions of C_1_–C_9_ hydrocarbons, which could help to further understand the origins and evolution of natural gas. Therefore, this experimental method can be successfully applied to analyze geological samples, thereby providing important information on the geochemical characteristics of natural gas, which would be very useful for geochemists.

## Data Availability

The data on content of hydrocarbons and repeatability of chemical composition that are used to support the findings of this study are included within the article. The data on extraction coefficient and carbon isotopic composition that are used to support the findings of this study are available from the corresponding author upon request.
